# Role of livestock in circular bioeconomy systems

**DOI:** 10.1093/af/vfaf022

**Published:** 2025-09-19

**Authors:** Tim A McAllister, Philippe Becquet, Barbara R Amon, LEAP TAG, Michael R F Lee

**Affiliations:** Lethbridge Research and Development Centre, Agriculture and Agri-Food Canada, Lethbridge, Alberta, Canada; Mulhouse, France; Leibniz Institute for Agricultural Engineering and Bioeconomy (ATB), Technology Assessment, Potsdam, Germany; University of Zielona Góra, Institute of Environmental Engineering, Zielona Góra, Poland; LEAP Technical Advisory Group; Harper Adams University, Office of the Deputy Vice-Chancellor, Newport, UK

**Keywords:** biomass, circular bioeconomy, greenhouse gas emissions, livestock, sustainability

**Implications**

- A circular bioeconomy integrates both bioeconomy principles and circular principles to create sustainable, low-impact solutions that ensure efficient use of biological resources.
- Livestock play an important role in a circular bioeconomy as they enable the upcycling of agricultural products unsuitable for consumption by humans into nutritionally rich animal-sourced foods, and their excrements serve as valuable organic fertilizer.
- Understanding positive and negative environmental impacts of livestock production systems is the key to establishing a sustainable circular bioeconomy.

## Introduction

The circular bioeconomy represents a transformative approach to sustainability by fostering the efficient use, reuse, and regeneration of renewable biomass (i.e., animals, plants, microorganisms, and their derived products). Implementation of a circular bioeconomy can offer solutions to address global challenges such as resource depletion, biodiversity loss, waste management, and environmental impacts, including climate change. To produce food, feed, materials, and energy, humans use biomass from both natural and managed ecosystems. Natural resources are the fundamental pillar of the food system, and the resulting production of biomass is pivotal to the development of a bioeconomy sector that enables the transition from fossil fuels to renewable energy (Muscat et al., 2021). However, current levels of biomass harvesting are associated with a variety of environmental issues such as land use, biodiversity loss, and climate change (Krausmann et al., 2013). As the global human population continues to grow, the demand for biomass increases, and these issues are exacerbated. To prevent further exceeding of the planetary boundaries, there is widespread acknowledgment of the need to transform our economy, including our food system in terms of production, consumption, and waste production (Steffen et al., 2015; Richardson et al., 2023). Promoting a circular bioeconomy that fits within planetary boundaries is widely recognized as one of the primary strategies to achieve this goal. A global repository of bioeconomy policies based on societal aspirations, good governance needs, and opportunities to valorize and protect biomass and scientific breakthroughs in biological, digital, and other fields has been developed to support sustainability and circularity (FAO, 2024).

## Circular Bioeconomy

The term bioeconomy has been defined as “the production, utilization, conservation and regeneration of biomass, including related knowledge, science, technology, and innovation to provide sustainable solutions (i.e., information, products, processes, and services) within and across all economic sectors to enable transformation to a sustainable economy” (International Advisory Council on Global Bioeconomy & Global Bioeconomy Summit, 2020). The bioeconomy involves sectors and interlinked systems that rely on biomass including terrestrial and marine ecosystems, primary production sectors (i.e., crop and livestock production, forestry, fisheries, and aquaculture), and all activities that use biomass to produce food, feed, fiber, energy, and other biobased products and services (Gomez San Juan et al., 2022). As a result, a circular bioeconomy offers a conceptual framework for using renewable natural capital to transform and manage land, food, health, and industrial systems, with the goal of achieving a sustainable well-being that is aligned with nature.

The bioeconomy addresses global, multidimensional challenges, but it is not inherently sustainable, as it risks perpetuating a linear economic model, one which favors short-term gain over long-term sustainability (Stegmann et al., 2020; FAO, 2021). A circular bioeconomy uses biomass more efficiently and strives to retain components (i.e., nutrients) within the system in a manner that promotes sustainability (Reichel et al., 2016) and the regeneration of natural and/or managed (eco)systems so as to reduce finite resource demand. Resource efficiency is promoted, while waste production is ideally reduced or eliminated (Reichel et al., 2016).

**Figure 1.**
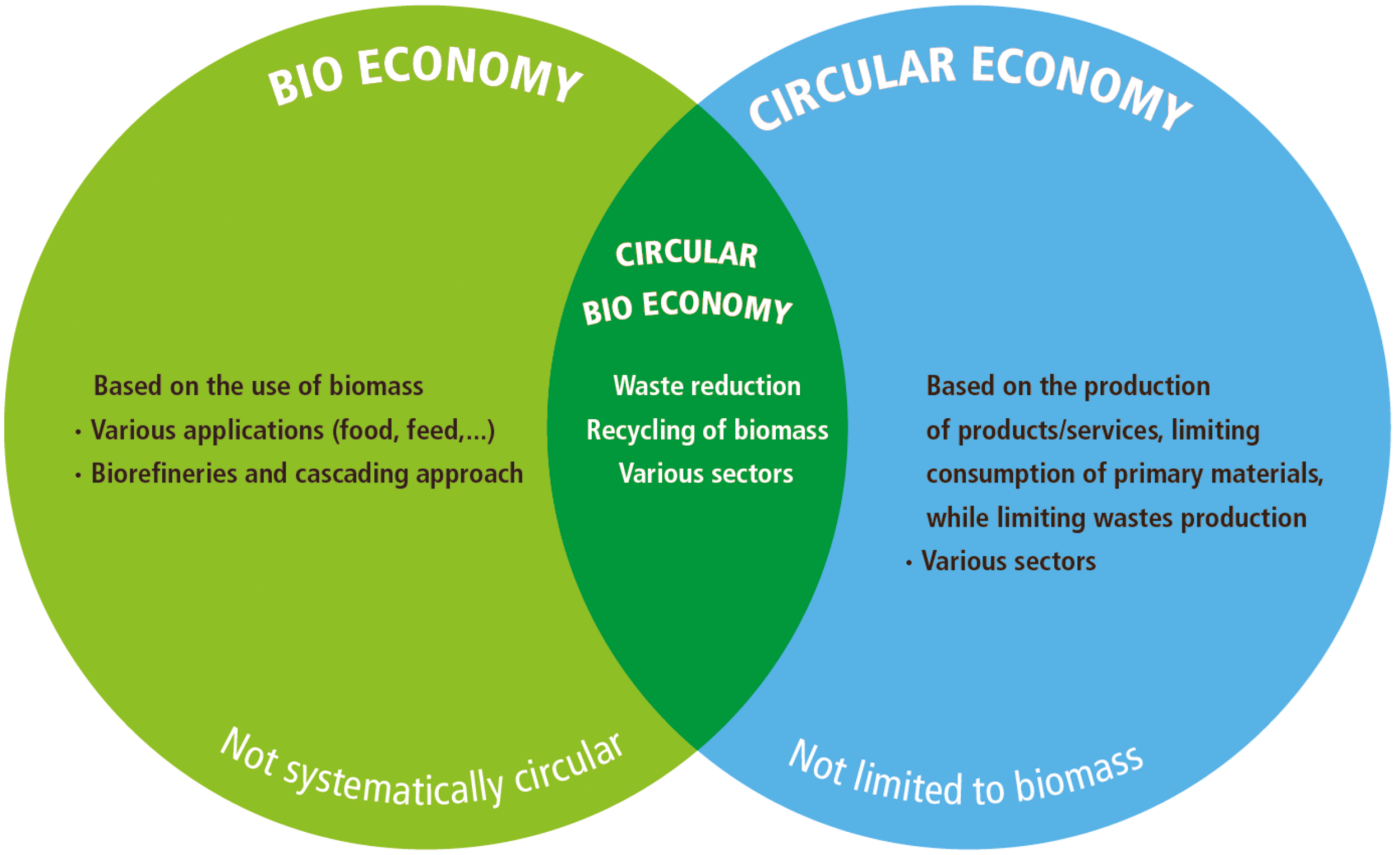
Bioeconomy and circular economy frameworks and their integration into circular bioeconomy.

A circular bioeconomy is at the intersection between the bioeconomy and the circular economy, with an emphasis on the sustainable use of biomass through closed-loop systems that rely on reducing, reusing, and recycling biomass (Figure 1). Hence, the circular bioeconomy provides ecosystem services that allow sustainable production, use, conservation, and regeneration of biomass and their transformation to food, feed, fiber, fuel, and other materials within ecosystem boundaries. It aims to support sustainable well-being for society at large, based on healthy, biodiverse, and resilient ecosystems (Palahi et al., 2020). Achieving a resource-efficient global circular bioeconomy is projected to generate USD $7.7 trillion by 2030 (WBCSD, 2019).

### Role of livestock on circular bioeconomy

Currently, many agricultural and livestock activities have components that are of an intrinsically linear nature. They involve the harvest of a certain amount of biomass from the system, where a large proportion of inputs do not contribute to products directly consumed by humans. This can generate losses and waste that, if not returned to the system, can have negative consequences for the environment. Under this paradigm, achieving circularity in the food system implies searching for practices and technologies that minimize the input of finite resources (e.g., fossil fertilizers and fuels, water, and land), encourage the use of regenerative practices, and stimulate reuse/recycling of residual streams (e.g., human and livestock excreta) in a manner that adds the highest value to unavoidable food system residues (Ghisellini et al., 2014; Jurgilevich et al., 2016; Corona et al., 2019; Valls-Val et al., 2023). Implementation of circularity in livestock systems should also consider the accessibility and ability to adopt the practice as well as its implications for animal health and well-being (Puente-Rodríguez et al. 2022). Impacts on broader society and how outcomes may be influenced by human behavior should also be considered (Corona et al. 2019). The livestock sector plays a key role in promoting circularity (e.g., upcycling, recycling, etc.), and the adoption of circular bioeconomy principles can improve the sustainability of livestock production. Effective implementation of a circular bioeconomy for livestock must also consider multiple ancillary sectors and value/supply chain components, such as transport, packaging, and storage, but the primary determinants of circularity are linked to feed utilization, manure management, and the utilization of livestock products.

**Figure 2.**
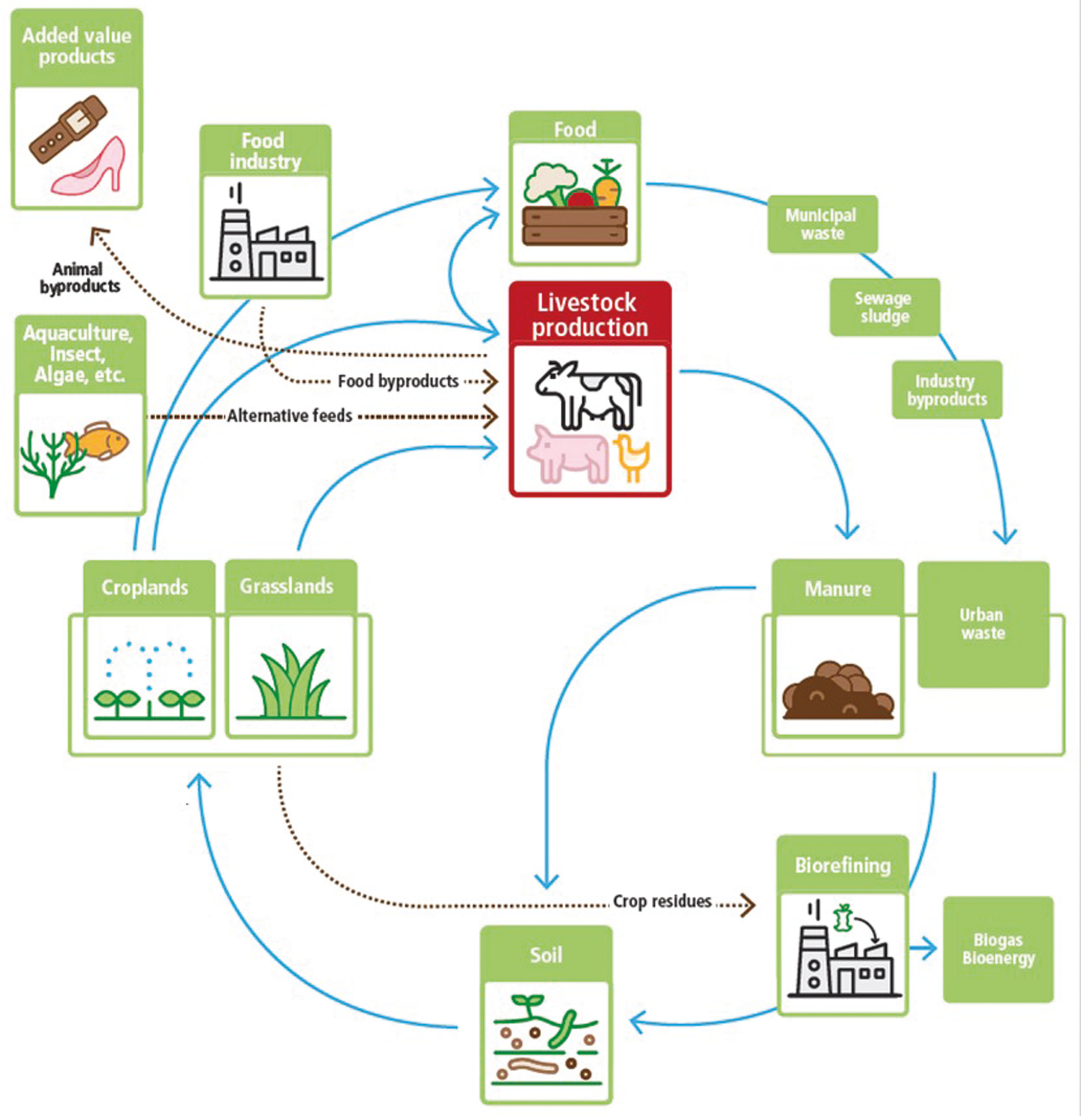
Representation of a circular bioeconomy system for livestock (van Zanten et al., 2019).

In a circular bioeconomy, arable land is used primarily to produce food and materials for other needs (De Boer and Van Ittersum, 2018; Van Zanten et al., 2019). During the production and consumption of food, residuals, and coproducts are generated from agricultural activities, industrial food processing, food losses and waste, and human and animal excreta. A principal priority is to prevent human edible byproducts from becoming food waste. Under this paradigm, livestock play a crucial role in circular bioeconomy by recycling resources that are not part of the primary food basket. This is accomplished through the production of food, utilization of human nonedible plant-based products (PBP) and animal-based byproducts (ABP), residual management, nutrient cycling, soil health, biodiversity, and renewable energy generation (Figure 2). Livestock are also essential to the sustainability of integrated crop-livestock systems, where the inclusion of forages in rotational cropping systems and the provision of manure contribute to carbon sequestration and soil health (Giacometti et al., 2021). Thus, livestock play an important role in the circular bioeconomy as they enable the upcycling of agricultural products that cannot be consumed by humans into valuable nutritional animal-sourced foods and produce manure as a fertilizer (Eisler et al., 2014). Livestock can also be used for drafting and delivering other ecosystem services and cultural values.

Animal-sourced foods provide a significant portion of the world’s food supply, including 34 to 40% of global protein consumption as well as the provision of vital micronutrients, which are more difficult to obtain from plant-based foods alone (FAO, 2023). By utilizing nonedible biomass such as grasslands, crop residues, crops designated unsuitable for food, and byproducts from other industries (e.g., oilseed meals), livestock can convert low-value resources into high-quality nutrient sources for humans. This promotes circularity while generating food, feed, and biomaterials. Using byproducts and food loss/waste as feed frees up land that can be better used to grow field and horticulture crops in support of human nutrition, as well as that which can be set aside to conserve biodiversity. Of the feed consumed by livestock, 86% is estimated to be unsuitable as food for humans, with the remaining 14% accounting for one-third of global cereal production (Mottet et al., 2017). Under a circular paradigm, food-feed competition is avoided, while livestock recycle residual streams from food-feed production and biobased industries.

Livestock also play a vital role in nutrient cycling and soil health, functions that are critical to sustainable agroecosystems. Livestock produce manure, which, if used effectively, can be a valuable organic fertilizer rich in macro and micronutrients and organic matter, although poor handling and application can result in pollution of air and watercourses. Many of the nutrients in manure are components of organic matter and, as a result, are released into the soil profile more slowly than their inorganic counterparts. This property can increase the likelihood of nutrient capture by crops and reduce the risk of ground or surface water contamination. Coupling crop and livestock production at adequate density, together with appropriate management of excreta as a nutrient source for crops, contributes to agricultural sustainability and reduces the need for synthetic fertilizers (Soussana and Lemaire, 2014). This closed-loop approach can help maintain soil fertility, promote soil health, increase nutrient cycling, enhance long-term crop productivity, reduce the need for expensive (economically and environmentally) synthetic fertilizers, and subsequently reduce production costs (Rufino et al., 2006).

Due to the linear nature of current industrial livestock and agricultural systems, not all system inputs contribute to products consumable by humans, and generated residuals have the potential to cause pollution (FAO, 2023). It is estimated that between USD $1 to 2 trillion per annum is lost through inefficiencies in the global food economy, and as much as 31% of the food produced for human consumption is wasted (UNEP, 2024). Livestock production systems rarely produce a single product, raising the possibility that one commodity can add value to another through circularity (e.g., food byproducts as feed for livestock or whey used to enhance fermentation in biobased industries).

**Figure 3.**
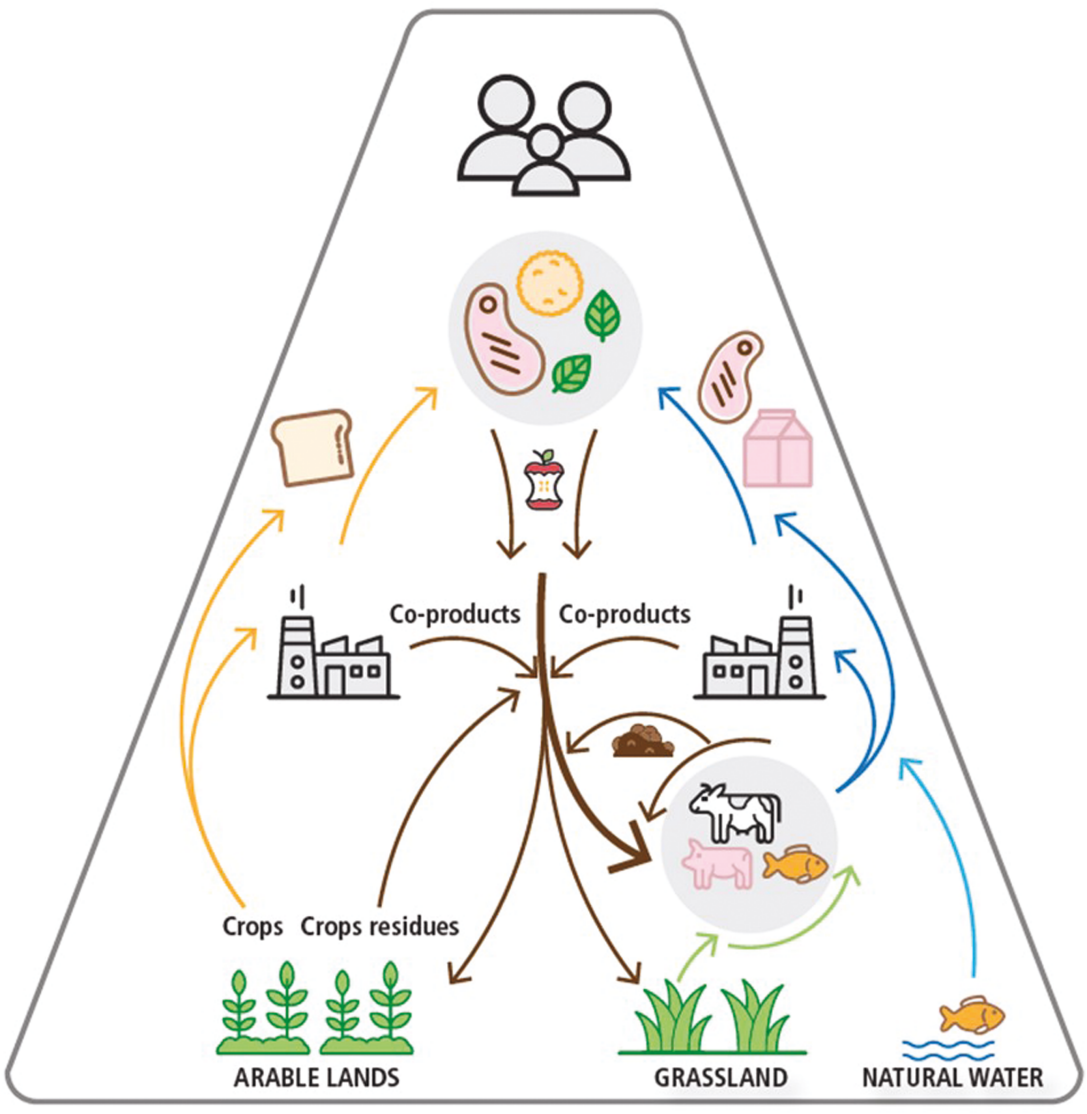
The biophysical concept of circularity. Arable land is primarily used for food production; biomass unsuited for direct human consumption is consumed by animals. Some coproducts and manure are used to maintain soil fertility. In this way, nutrients are recycled, and animals contribute to circularity and improve the sustainability of the food system (Van Zanten et al., 2019).

Livestock can recycle and upcycle resources while playing an important role in feeding humanity by consuming low opportunity cost byproducts (LCB) and biomass from grasslands. In a circular bioeconomy, livestock are fed biomass unsuitable for consumption by humans; thereby producing valuable animal-sourced foods, animal byproducts (e.g., leather and wool), manure, and other ecosystem services (Figure 3). Available biomass to feed livestock includes crop residues, forages produced on lands less suitable for the cultivation of food crops, byproducts arising from the industrial processing of PBP, ABP, biofuels, fermentation products, as well as food loss and residuals unsuitable for human consumption. By converting these LCB streams, livestock recycle nutrients back into the food system that otherwise would be lost. As a result, the food-feed competition for land is reduced (Van Zanten et al., 2018; Wilkinson and Lee, 2018).

#### Livestock and land.

Land used for agriculture constitutes about 38% of the global land area, with one-third of this dedicated to crop production and the remaining two-thirds utilized by grazing livestock (FAO, 2020). Of available cropland, 40% is used to produce high-quality feed ingredients, with the remaining used for animal feed and other purposes (e.g., energy crops and fiber), resulting in a food-feed-fiber-energy competition for land and other natural resources. As livestock require energy for maintenance, they consume more calories from feed than they produce as muscle, milk, or eggs. Unlike carbohydrate-rich plant stables and oil seeds, which are consumed primarily as a source of energy, animal-sourced foods are consumed as a source of protein and highly digestible and bioavailable micronutrients (minerals and vitamins). The food-feed competition can be direct or indirect. Direct competition occurs when biomass suitable for human consumption is fed to livestock instead of humans, often because the food has failed to meet quality criteria or value-chain constraints (Wilkinson and Lee, 2018). Indirect competition occurs when feeds are cultivated in areas where crops for human consumption could be grown (van Zanten et al., 2016a).

Food vs. biomass for biofuel competition also occurs, but unlike livestock production, biofuel production does not produce products that can be consumed by humans (Prasad and Ingle, 2019). Although other sources of renewable energy, such as wind and solar, also occupy land, areas within wind and solar farms can still be used for food production. In both biofuel and livestock production, land ends up being purposed to produce biomass instead of food for humans. Some livestock, such as pigs and poultry, rely more on arable land for feed production, whereas ruminants (e.g., sheep and cattle) may derive nutrients by grazing lands that are less suitable for producing food for humans (Lee et al., 2021). In terms of global food production, feeding animals more LCB could significantly increase the global food supply by freeing up land that could be used to produce food for humans as opposed to feed for livestock. Sandström et al. (2022) estimated that if whole fish, pulses, and vegetable oil were replaced with food system byproducts, global food availability would increase by 13% and 15% in terms of kilocalories and protein, respectively. However, such an approach could possibly be at the cost of a reduction in livestock productivity if diets are unbalanced or of lower nutritional quality. While more kilocalories and proteins do not necessarily result in improved nutrition, they could contribute to improved food security in many parts of the world. Redesigning the livestock sector based on circularity principles offers the opportunity to reduce food-feed competition, lower environmental impacts, improve the efficiency of water, energy, and natural resource use while contributing to global food security.

#### Role of livestock manure.

Circular bioeconomy principles are promoted by environmentally friendly manure management practices. Manure from livestock is a valuable organic fertilizer that can be used to replace a portion of synthetic fertilizer and serve as a source of biomass for bioenergy generation (Arsic et al., 2025). Anaerobic digestion of manure can generate biogas, a renewable energy source primarily composed of methane (CH_4_). Biogas can be harnessed to produce heat and electricity or further purified to CH_4_ for injection into natural gas grids or liquified for use as a transportation fuel. Converting manure into biogas simultaneously addresses residual management challenges, reduces GHG emissions, and provides a renewable energy resource that lowers reliance on conventional fossil fuels. Furthermore, the process produces a nutrient-rich digestate, which can be utilized as a fertilizer or subject to further refinement (Dubis et al., 2022).

#### Animal byproducts.

**Figure 4.**
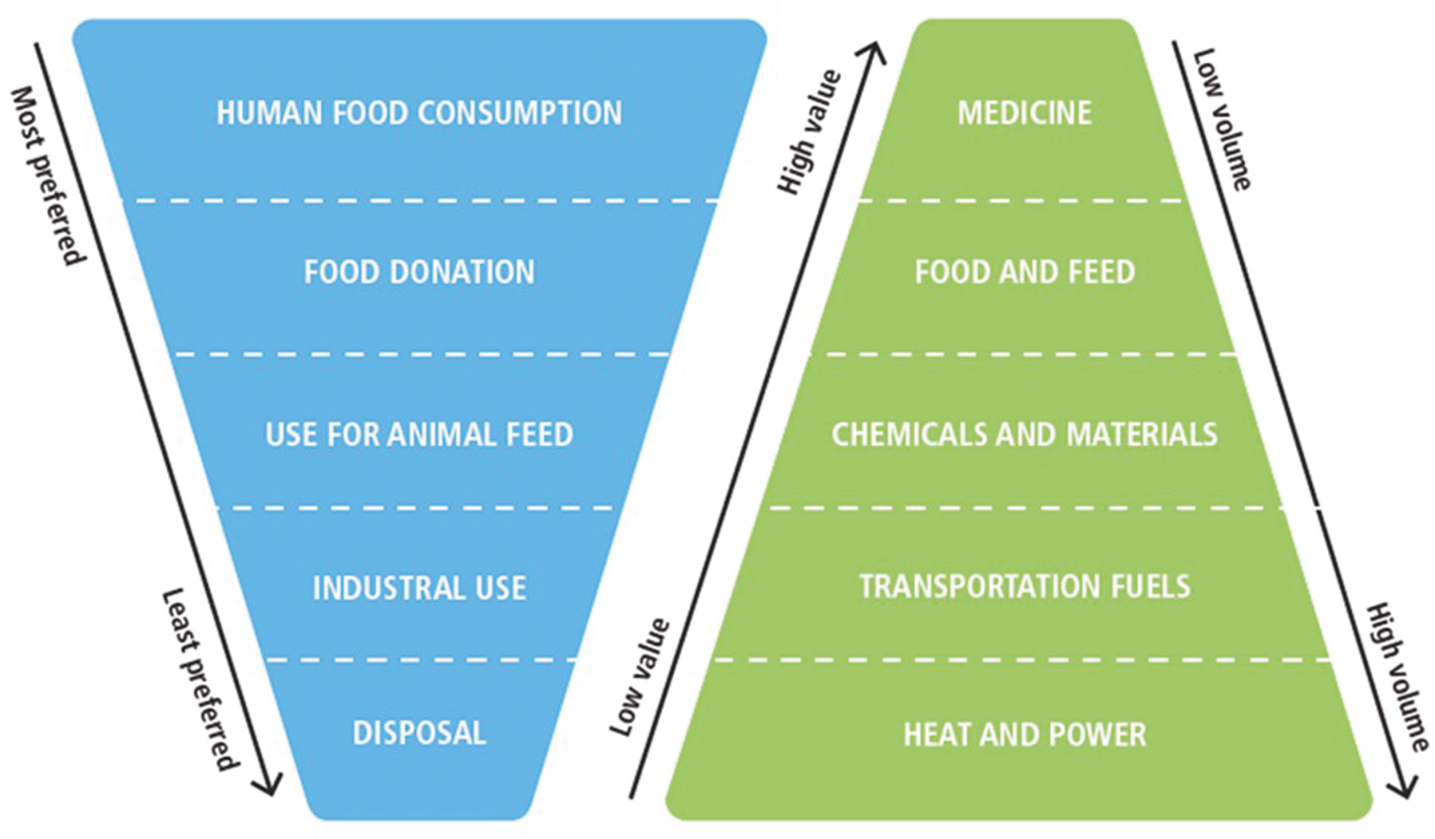
Animal-based products from the abattoirs hierarchy and value pyramid (Al-Zohairi et al., 2023).

Animal byproducts, including bones, hides, and offal, have numerous applications beyond traditional food production (Lee et al., 2025). These materials can be utilized to produce value-added products such as leather, gelatin, pet food, livestock feed, pharmaceuticals, cosmetics, and biodiesel. Moreover, many of these are rich in collagen, keratin, and minerals, making them a source of high-value biochemicals and other biomaterials that are employed in various industrial applications. The concept of valorization of ABP follows the principle of the “Food Waste Hierarchy” and “Value Pyramid” described by Al-Zohairi et al., (2023; Figure 4). Sometimes these concepts are described as a “cascading use of biomass” (Dubois and Gomez San Juan, 2016). The preferred option is source prevention (i.e., avoiding generation of food waste), followed by food recovery, where a greater proportion of plant or animal biomass is used or recovered as human edible food. After food recovery, the value pyramid proposes recycling of byproducts to produce food through their use as feed, followed by recovery of byproducts for industrial applications. This can include the production of a range of byproducts such as pet foods, lower-value chemicals, and materials such as fertilizers, soap, or biodiesel. Animal-based products can also be used as a substrate for biodigestion, and when all alternative uses have been exhausted, ABP can be combusted to generate energy.

### Livestock in a circular bioeconomy and sustainable development goals

The 193 members of the FAO 2022–2031 Strategic Framework have made the bioeconomy a priority program area. While the bioeconomy contributes to all Sustainable Development Goals (SDGs), principles of sustainable consumption and production are front and center in FAO’s bioeconomy mandate. This involves using natural resources more sustainably, reducing pollution, and repurposing unavoidable waste. Although the bioeconomy advances SDGs (Calicioglu and Bogdanski, 2021), its outcomes are not necessarily sustainable. For governments to formulate policies and strategies that support a sustainable bioeconomy that includes livestock, they must promote an environment for biobased research, technological innovation, education, capacity building, industrialization, inclusive development in rural and urban areas, consumer demand creation, and enhanced societal awareness. Such an approach involves the identification of the inevitable trade-offs that will occur among SDGs (FAO, 2021). While every country has the potential to integrate livestock into its circular bioeconomy, not all have strategies to enhance sectoral synergies and address resource competition and sustainability trade-offs. FAO has published a framework that links 10 aspirational principles and 24 criteria for a sustainable bioeconomy to the SDGs to identify the optimal trade-off balance within circular bioeconomies. Central to FAO’s bioeconomy efforts, these principles encourage a comprehensive approach, integrating social, economic, and environmental dimensions of sustainability with good governance (FAO, 2018). Designed for policymakers and stakeholders, these principles can be used to define the role of both small and large-scale livestock stakeholders in bioeconomy policies and sustainability assessments.

Given that each country and region possesses distinct biomass use challenges and opportunities and goals shaped by their political, economic, industrial, and technological status, as well as their natural resources and societal characteristics, a universal solution to integrate livestock into a circular bioeconomy is nonexistent. Instead, tailored approaches are necessary to address the specific needs and circumstances of different countries and regions. Regarding circular bioeconomy systems in the livestock sector, the FAO has mapped unavoidable waste that can be used as by-products, identified plants that grow on marginal land or that require less fertilizer and water (FAO, 2022). Other approaches include insect-based feed ingredients, microbiome science from a “One Health” perspective, and improved breeding and management practices that reduce undesirable environmental impacts of livestock production, while generating socioeconomic benefits to communities.

## Conclusions

It is recognized that the livestock sector plays an important socioeconomic role in supporting sustainability through circularity, a practice that is frequently more developed in low than high-income countries (Paul et al., 2020). By promoting the use of biomass and energy through the reuse and recycling of residuals, a circular bioeconomy aims to reduce the consumption of finite resources and make biomass production more efficient. Livestock play a key role in promoting circularity within agricultural systems, and it is imperative that the synergies, trade-offs, and interactions that livestock play within a circular bioeconomy are well defined. Only in this manner will the contribution of livestock to transforming agricultural systems from linear “*take – make – utilize -waste*” to those that retain components and promote circular agricultural systems.

## About the Authors

**Tim A. McAllister** received an M.Sc. in Animal Biochemistry from the University of Alberta in 1987 and a Ph.D. in Microbiology and Nutrition from the University of Guelph in 1991. He has worked as a research scientist with Agriculture and Agri-Food Canada since 1996, where he now holds the position of principal research scientist in microbiology and beef cattle production. His team has addressed a variety of topic areas related to optimizing the role of livestock in a circular bioeconomy. These include assessing and mitigating greenhouse gas emissions from agricultural systems, optimizing manure management, utilizing food loss and waste as feed, promoting food safety, and recognizing the role of grassland ecosystems in sustaining biodiversity. His team has been the recipient of numerous societal awards for their contribution to beef cattle production in North America.

Corresponding autbor: Tim.McAllister@agr.gc.ca

**Philippe Becquet** is a consultant for regulatory and sustainability topics. He provides advice to the feed industry. Agronomist and animal nutritionist by background, Philippe Becquet has worked for more than 40 years in the feed industry at various positions and in various companies, accumulating experience and expertise in livestock production systems and the feed industry. He has also been active in feed industry associations such as IFIF (International Feed Industry Federation) and has contributed as an expert in the development of the Livestock Environmental Assessment and Performance (LEAP) guidelines on feed additives, the report on methane and more recently to the report on the role of livestock in circular bioeconomy.

**Barbara Amon** is an Associate Professor for Environmental Engineering and Agricultural Engineering at the University of Zielona Góra, Poland, and board representative for research at the Leibniz Institute for Agricultural Engineering and Bioeconomy in Potsdam, Germany. Having had many years of practical, hands-on experience in agriculture alongside extensive research experience, she completed her habilitation in Agricultural Engineering at the University of Natural Resources and Life Sciences in Vienna in 2007. In addition to her research, she sits on many panels looking at sustainable agriculture, including the Intergovernmental Panel on Climate Change, UN Environment Programme, and the FAO LEAP partnership. She is also the Co-Chair of the Agriculture and Nature Panel as part of the UNECE Task Force on Emission Inventories and Projections and of the Expert Panel on Mitigation of Agricultural Nitrogen under the UNECE Task Force on Reactive Nitrogen.

**Michael R.F. Lee** is an expert in sustainable livestock systems, defining their role in securing global food security at the same time as protecting environmental health (Livestock’s role in human and planetary health). He graduated with first-class honors in Animal Science from the University of Wales, Aberystwyth in 1997 and gained a PhD in ruminant nutrition (protein and energy metabolism) from the University of Aberdeen in 2001, followed by a Postgraduate Certificate for teaching in higher education from Aberystwyth University in 2012. In November 2020, Michael moved from Rothamsted Research and the University of Bristol (where he held a joint position) to his current position as Deputy Vice Chancellor of Harper Adams University, England’s Premier specialist agriculture and land use University. He has published over 375 research articles and papers, including articles in Nature and Science. He served as President of the British Society of Animal Science from 2018 to 2022 and was a Trustee of the society for 12 years. In August 2019, he was elected President of the European Federation of Animal Science Livestock Farming Systems Commission. He represents the UK on the Animal Task Force, a Public–Private think-tank informing the EU parliament in Brussels, and is co-chair of the UK Universities Climate Network—Net Zero group. In 2023, he was selected to join a Technical Advisory Group of the UN’s Food and Agriculture Organization (FAO). Prof Lee also sits on the BBC’s Rural Affairs Committee, the Agriculture Advisory Group convened by the UK’s Climate Change Committee (CCC), and the Scientific Council of the World Farmers Organization (WFO).

## LEAP Technical Advisory Group (LEAP TAG)

Luiz Abdalla Adibe (University of São Paulo, Brazil), Dagoberto Arias Aguilar (Instituto Tecnológico de Costa Rica, Costa Rica), Maja Arsic (Commonwealth Scientific and Industrial Research Organization, Australia), Ana Paula Contador Packer (Empresa Brasileira de Pesquisa Agropecuária, Brazil), Ellen Dierenfeld (World Wildlife Fund, United States of America), Hongmin Dong (Chinese Academy of Agricultural Sciences, China), Maria Candela Garcia de Andina (National Institute of Industrial Technology, Argentina), Fernanda Garcia Sampaio (Food and Agriculture Organization - NFIAT, Italy), Carlos Gomez (Universidad Nacional Agraria La Molina, Peru), Marta Gomez San Juan (Food and Agriculture Organization, Rome), Eleazar Ubaldo Gonzalez (Lincoln University, United States of America), Arnoldo Gonzalez Reyna (Autonomous University of Tamaulipas, Mexico), Ammar Hajer (University of Carthage, Tunisia), Margaret Jewell (Meat & Livestock Australia, Australia), Maryline Kouba (International Feed Industry Federation, France), Bruno Lanfranco (National Institute of Agricultural Research of Uruguay, Uruguay), Stewart Francis Ledgard (AgResearch, New Zealand), Laurence Loyon (The Institut national de la recherche agronomique, France), Bernard Lukuyu Adubwa (International Livestock Research Institute, Uganda), Jesus Mendez Batan (Spain), David Meo Zilio (Consiglio per la Ricerca in Agricoltura, Italy), Griselda Asuncion Meza Ocampos (Universidad Nacional de Asunción, Paraguay), George Wamwere Josiah Njoroge (Livestock Value Chains at the International Livestock Research Institute, Kenya), Guillermo Pardo Nieva (Basque Centre for Climate Change, Spain), Chayan Kumer Saha (Bangladesh Agricultural University, Bangladesh), Saheed Salami (Mootral Limited, United Kingdom), Buchun Si (China Agricultural University, China), Mugabekazi Sylvie (Cleaner Production and Climate Innovation Centre, Rwanda), Jogeir Toppe (Food and Agriculture Organization of the United Nations - NFIMF, Italy), Hannah van Zanten (Wageningen University and Research, Netherlands), Peter Wanyama (Ministry of Science Technology and Innovations, Uganda), Els Willems (Agrifirm, Netherlands).
